# Validation of the Portable Next-Generation VECTRA H2 3D Imaging System for Periocular Anthropometry

**DOI:** 10.3389/fmed.2022.833487

**Published:** 2022-03-11

**Authors:** Wanlin Fan, Yongwei Guo, Xiaoyi Hou, Jinhua Liu, Senmao Li, Sitong Ju, Philomena Alice Wawer Matos, Michael Simon, Alexander C. Rokohl, Ludwig M. Heindl

**Affiliations:** ^1^Department of Ophthalmology, Faculty of Medicine and University Hospital Cologne, University of Cologne, Cologne, Germany; ^2^Eye Center, Second Affiliated Hospital, Zhejiang University School of Medicine, Hangzhou, China; ^3^Center for Integrated Oncology (CIO), Aachen-Bonn-Cologne-Duesseldorf, Cologne, Germany

**Keywords:** three-dimensional anthropometry, portable stereophotogrammetry devices, validity, reliability, periocular morphology

## Abstract

**Purpose:**

Portable three-dimensional imaging systems are becoming increasingly common for facial measurement analysis. However, the reliability of portable devices may be affected by the necessity to take three pictures at three time points. The purpose of this study was to evaluate the effectiveness of portable devices for assessing the periocular region.

**Methods:**

In 60 Caucasian volunteers (120 eyes), four facial scans (twice for each instrument) using the portable VECTRA H2 and static VECTRA M3 devices were performed; patients' heads were kept straight, looking ahead, with a neutral facial expression. One assessor set 52 periocular landmarks in the periocular area of each image and subsequently assessed intra- and inter-device reliability by comparing two within-device measurements and one between-device measurement, respectively.

**Results:**

The mean absolute difference (MAD) (0.13 and 0.12 units), relative error of measurement (REM) (0.61 and 0.68%), technical error of measurement (TEM)(1.02 and 0.80 units), relative TEM (rTEM) (5.51 and 4.43%), and intraclass correlation coefficient (ICC) (0.89, 0.89) showed good intra-device reliability for M3 and H2; MAD (0.63, 0.62 units), REM (2.83, 2.69%), TEM (1.31, 1.10 units), rTEM (7.62, 5.57%), and ICC (0.79, 0.83) indicated that inter-device reliability deteriorated compared to intra-device reliability and that the inter-device reliability of the first scan (moderate) was lower than that of the average of the two scans (good).

**Conclusions:**

The portable VECTRA H2 device proved reliable in assessing most periocular linear distances, curve distances, and angles; some improvement in inter-device reliability can be achieved by using the average of two scans.

## Introduction

The anthropometric data of facial soft tissues are widely used in plastic (1) and craniomaxillofacial surgery (2–4). These data are important to develop surgical plans (5) and assess outcome prognosis (6–8). Particularly, the periocular region plays an important role in facial attractiveness, emotional expression, and differentiation by ethnicity (9, 10), gender, and age (11, 12). It is also a major reference indicator for corrective, restorative, or cosmetic surgery (13). In recent years, non-invasive three-dimensional (3D) surface imaging methods, including VECTRA, Artec EVA, and 3D MD systems (14–16) have gradually replaced traditional direct anthropometric techniques (using rulers and calipers) and two-dimensional (2D) photography. Most of the existing 3D photogrammetry systems are static devices, which prominently feature in capturing photos of participants from three different angles at a single time point and composing a 3D photo using a computer. Previous studies have proposed the first standardized periocular anthropometric protocol (17, 18) and showed potential clinical applications, including a novel standardized lower eyelid tension distraction test and a lateral distraction test (19, 20). The reliability (repeatability) and accuracy of the VECTRA M3 static device is very high for linear, curvilinear, angular, area, and volume measurements (21–25). However, the device is expensive, bulky, untransportable, and requires frequent calibration (2, 14, 26–28) to reach this high reliability, which are considerable limitations, especially for patients who cannot walk independently or reside in remote and poor areas.

Currently, portable 3D imaging devices are available in the market. These systems comprise only one digital single-lens reflex camera in addition to a computer system (5). Due to low cost, no need for calibration, and portability, portable 3D photogrammetry systems have high potential to be used extensively in research and routine clinical measurements in the future. Although several publications have conducted facial analyses using older portable devices (5, 16, 29–31), including studies on the reproducibility of these devices (VECTRA H1) in comparison with static devices (3D MD or VECTRA M3) (5, 31), some issues remain to be addressed. First, the primary portable device used in previous studies was the VECTRA H1; the newest generation, VECTRA H2, was not used. Second, there are no studies on the application of portable devices in the periocular area with newly developed standardized landmarks protocols. Finally, many factors, including head and eye movements, camera movements, user dependence, and facial expressions, may affect the reliability of the portable device during the three shots. Therefore, this study aimed to evaluate the reliability of a novel portable stereophotogrammetric device VECTRA H2 compared to the static VECTRA M3 (the current gold-standard 3D imaging system) for three-dimensional periocular analysis and subsequently provide a basis for the feasibility of VECTRA H2 in periocular applications.

## Methods

### Study Participants

Sixty Caucasian volunteers (30 men and 30 women, 120 eyes) aged 18–48 years (28.2 ± 6.2 years) were recruited for this study. The study sample size was calculated based on the results of the interdevice comparison between M3 and H2 for 10 volunteers in the pre-test study (LCAm: 67.77 ± 10.65**°** vs. 62.98 ± 7.09**°**). With a 2-sided 5% significance level and 80% power, a sample size of 34 patients per group was determined by PASS software (Version 15, UT, USA). Exclusion criteria were deformities, lesions, surgical, or traumatic events involving the face. All participants signed an informed consent form, and this study was performed in accordance with the principles of the Declaration of Helsinki and approved by the ethics committee of Cologne University (approval no: 17-199).

### 3D Image Acquisition and Data Collection

**Figure 1:**
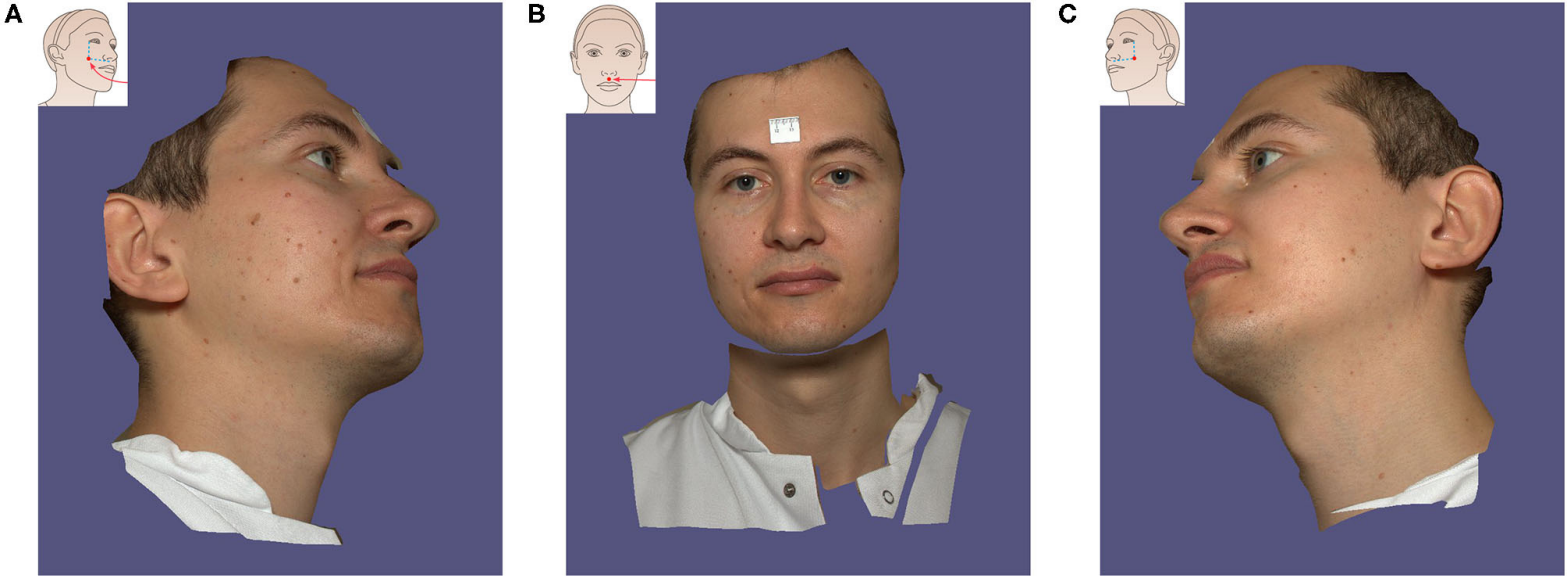
The process of 3D image acquisition with Vectra H2 portable camera. The volunteer follows the instructions to turn the head to the left **(A)**, turn the head to look forward **(B)**, and finally turn the head to the right **(C)**. The application instruction screen for each step is displayed in the upper right corner.

Before the images were obtained, all volunteers were asked to remove their makeup, take off their jewelry, and pull their hair back to ensure complete exposure of their forehead and eyebrows. Thereafter, the facial images of each volunteer were captured twice by a static VECTRA M3 and a portable VECTRA H2 system (Canfield Scientific, Inc., Parsippany, NJ, USA). Scanning with both devices was performed consecutively in the same room, and the volunteers sat in a neutral posture. For the static VECTRA M3, calibration was performed according to device guidance before each capture. During acquisition, participants looked at the upper-middle mirror in the machine, keeping their eyes between the vertical and horizontal reference lines on the screen. For the portable VECTRA H2, the operator took three consecutive photographs from three angles as required by the device instructions: the first photograph was taken 30 cm below 45 degrees on the right side of the volunteer's face, and the second photograph was taken with the camera in front of the face. Subsequently, the third picture was taken on the left side of the face at 30 cm below 45 degrees (Figure 1). Finally, the computer connected to the camera merged the three photos into one 3D photo using VAM software version 2.8.2 (Canfield Scientific, Inc.).

**Figure 2:**
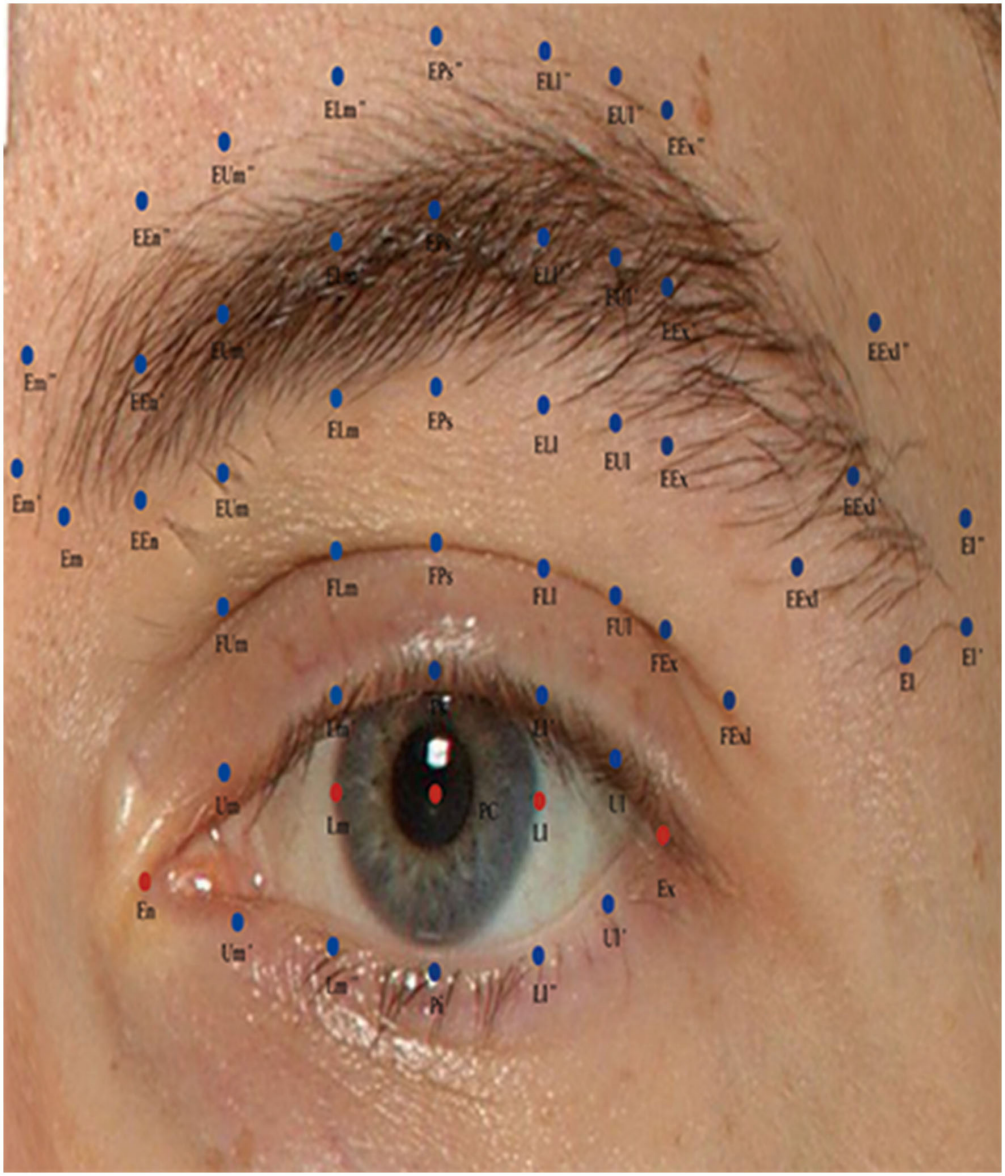
Description of periocular anthropometric landmarks used in this study. Periocular anthropometry was performed according to Guo et al. (17).

This study employed 52 3D anthropometric landmarks of the periocular region developed and validated by our research group (Figure 2). The definitions of these landmarks and measurements are detailed in Tables 1, 2. Subsequently, the study measured three categories of data (linear distances, curves, and angles).

**Table 1:** Definition of abbreviations for periocular landmarks, modified from Guo et al. (17).

**Abbreviation of landmarks**	**Definition**
En	Endocanthion, inner commissure of the palpebral fissure
Ex	Exocanthion, outer commissure of the lower and upper eyelash roots of the palpebral fissure
Pc	Pupillary center
Lm	Medial corneoscleral limbus point horizontal to pupillary center
Ll	Lateral corneoscleral limbus point horizontal to pupillary center
Em	Inferior margin point of the medial eyebrow end (sometimes locates at the same place with EEn);
Em”	superior margin point
Em'	middle point
EEn	Inferior margin point of eyebrow vertical to En
EEn”	Superior margin point of eyebrow vertical to En
EEn'	Middle point of eyebrow vertical to En
Um	Middle point between En and Lm' at the upper palpebral margin on the lash roots
Um'	Middle point between En and Lm” at the lower palpebral margin on the lash roots
FUm	Point vertical to Um at the lid fold superioris
EUm	Point vertical to Um at the inferior margin of eyebrows
EUm”	Point vertical to Um at the superior margin point
EUm'	Point vertical to Um at the middle point
Lm'	Point vertical to Lm at the upper palpebral margin on the lash roots
Lm”	Point vertical to Lm at the lower palpebral margin on the lash roots
FLm	Point vertical to Lm at the lid fold superioris
ELm	Point vertical to Lm at the inferior margin of eyebrows
ELm”	Point vertical to Lm at the superior margin of eyebrows
ELm'	Point vertical to Lm at the middle margin of eyebrows
Ps	Palpebrale superioris, Point vertical to Pc at the upper palpebral margin on the lash roots
Pi	Palpebrale inferioris, Point vertical to Pc at the lower palpebral margin on the lash roots
FPs	Point vertical to Pc at the lid fold superioris
EPs	Point vertical to Pc at the inferior margin of eyebrows
EPs”	Point vertical to Pc at the superior margin of eyebrows
EPs'	Point vertical to Pc at the middle margin of eyebrows
Ll'	Point vertical to Ll at the upper palpebral margin on the lash roots
Ll”	Point vertical to Ll at the lower palpebral margin on the lash roots
FLl	Point vertical to Ll at the lid fold superioris
ELl	Point vertical to Ll at the inferior margin of eyebrows
ELl”	Point vertical to Ll at the superior margin of eyebrows
ELl'	Point vertical to Ll at the middle margin of eyebrows
Ul	The middle between Ex and Ll' at the upper palpebral margin on the lash roots
Ul'	The middle between Ex and Ll” at the lower palpebral margin on the lash roots
FUl	FUl Point vertical to Ul at the lid fold superioris
EUl	Point vertical to Ul at the inferior margin of eyebrows
EUl”	Point vertical to Ul at the superior margin of eyebrows
EUl'	Point vertical to Ul at the middle margin of eyebrows
FEx	Point vertical to Ex at the lid fold superioris
EEx	Point vertical to Ex at the inferior margin of eyebrows
EEx”	Point vertical to Ex at the superior margin of eyebrows
EEx'	Point vertical to Ex at the middle margin of eyebrows
FExl	Point vertical to Ex at the lid fold superioris in lateral view
EExl	Point vertical to Ex at the inferior margin of eyebrows in lateral view
EExl”	Point vertical to Ex at the superior margin of eyebrows in lateral view
EExl'	Point vertical to Ex at the middle margin of eyebrows in lateral view
El	inferior margin of the lateral eyebrow end
El”	superior margin of the lateral eyebrow end
El'	middle margin of the lateral eyebrow end

**Table 2:** List of linear distance, curve, and angle measurement variables for the periocular region, derived from Guo et al. (17).

**Abbreviation**	**Definition**	**Landmarks**
**Liner distances**		
PFW	Palpebral fissure width	En-Ex
PFH	Palpebral fissure height	Ps-Pi
EEnD_I	Eyebrow-endocanthion distance of the inferior point	EEn-En
EEnD_M_	Eyebrow-endocanthion distance of the middle point	EEn'-En
EEnD_S_	Eyebrow-endocanthion distance of the inferior, middle, or superior point	EEn”-En
FPDm	Upper lid fold-palpebral margin distance (medial)	FUm-Um
EPDm_I	Eyebrow-palpebral margin distance (medial) of the inferior point	EUm-Um
EPDm_M	Eyebrow-palpebral margin distance (medial) of the middle point	EUm'-Um
EPDm_S	Eyebrow-palpebral margin distance (medial) of the superior point	EUm”-Um
FLmD	Upper lid fold-palpebral margin distance (medial limbus)	FLm-Lm'
ELmD_I	Eyebrow-palpebral margin distance (medial limbus) of the inferior point	ELm-Lm'
ELmD_M	Eyebrow-palpebral margin distance (medial limbus) of the middle point	ELm'-Lm'
ELmD_S	Eyebrow-palpebral margin distance (medial limbus) of the superior point	ELm”-Lm'
FPD	Upper lid fold-palpebral margin distance, similar to upper lid fold height	Ps-FPs
EPD_I	Eyebrow-palpebral margin (Ps) distance of the inferior (similar to upper lid height) point	Ps-EPs
EPD_M	Eyebrow-palpebral margin (Ps) distance of the middle point	Ps-EPs'
EPD_S	Eyebrow-palpebral margin (Ps) distance of the superior point	Ps-EPs”
FLlD	Upper lid fold-palpebral margin distance (lateral limbus)	FLl-Ll'
ELlD_I	Eyebrow-palpebral margin distance (lateral limbus) of the inferior point	ELl-Ll'
ELlD_M	Eyebrow-palpebral margin distance (lateral limbus) of the middle point	ELl'-Ll'
ELlD_S	Eyebrow-palpebral margin distance (lateral limbus) of the superior point	ELl”-Ll'
FPDl	Upper lid fold-palpebral margin distance (lateral)	FUl-Ul
EPDl_I	Eyebrow-palpebral margin distance (lateral) of the inferior point	EUl-Ul
EPDl_M	Eyebrow-palpebral margin distance (lateral) of the middle point	EUl'-Ul
EPDl_S	Eyebrow-palpebral margin distance (lateral) of the superior point	EUl”-Ul
FExD	Upper lid fold-exocanthion distance	FEx-Ex
EExD_I	Eyebrow-exocanthion distance of the inferior point	EEx-Ex
EExD_M	Eyebrow-exocanthion distance of the middle point	EEx'-Ex
EExD_S	Eyebrow-exocanthion distance of the superior point	EEx”-Ex
FExDl	Upper lid fold-exocanthion distance (lateral)	FExl-Ex
EExDl_I	Eyebrow-exocanthion distance (lateral) of the inferior point	EExl-Ex
EExDl_M	Eyebrow-exocanthion distance (lateral) of the middle point	EExl'-Ex
EExDl_S	Eyebrow-exocanthion distance (lateral) of the superior point	EExl”-Ex
ID	Iris diameter	Lm-Ll
EnD	Inner intercanthal distance	En (left)-En (right)
PD	Interpupillary distance	Pc (left)-Pc (right)
ExD	Outer intercanthal distance	Ex (left)-Ex (right)
**Curvatures**		
UPML	Upper palpebral margin length	En-Um-Lm'-Ps-Ll'-Ul-Ex
UPMLm	Upper palpebral margin length (more points)	Including 4 more midpoints between Lm'-Ps-Ll'-Ul-Ex
LPML	Lower palpebral margin length	En-Um'-Lm”-Pi-Ll”-Ul'-Ex
LPMLm	Lower palpebral margin length (more points)	Including 4 more midpoints between Lm”-Pi-Ll”-Ul'-Ex
EL_I	Inferior eyebrow length	Em-EEn-EUm-ELm-EPs-ELl-EUl-EEx-EExl-El
EL_M	Middle eyebrow length	Em'-EEn'-EUm'-ELm'-EPs'-ELl'-EUl'-EEx'-EExl'-El'
EL_S	Superior eyebrow length	Em”-EEn”-EUm”-ELm”-EPs”-ELl”-EUl”-EEx”-EExl”-El”
**Angles**		
MCA	Medial canthal angle	Ps-En-Pi
MCAm	Medial canthal angle (medial)	Um-En-Um'
LCA	Lateral canthal angle	Ps-Ex-Pi
LCAm	Lateral canthal angle (medial)	Ul-Ex-Ul'
CT	Canthal tilt	Ex (left)-En (left)-En (right), or Ex (right)-En (right)-En (left)

### Statistical Analysis

Statistical analysis was performed using SPSS 23.0 software (Armonk, NY: IBM Corp.), and graphs were created by GraphPad Prism 9 (GraphPad Software, San Diego, CA, USA). Differences in the age distribution of men and women among volunteers were assessed using the Wilcoxon's signed rank-sum test. All measured data were tested for normality using the Kolmogorov-Smirnov test. For data conforming to a normal distribution, paired *t*-tests were conducted to assess differences within and between devices. For non-normally distributed data, Wilcoxon's signed-rank test was used. *P*-values <0.05 were considered to indicate statistical significance.

Intra-device reliability was analyzed by comparing the images captured twice by each device (VECTRA M3 and VECTRA H2), and inter-device reliability was analyzed by comparing the metric parameters obtained from the first scan (using VECTRA H2 and VECTRA M3) and the measured average of the images scanned twice with each device. The intraclass correlation coefficient (ICC) has a value between 0 and 1, and a value closer to 1 indicates high reliability. ICC values allowed the classification of the agreement into three classes: <0.4, poor agreement; 0.4–0.75, satisfactory; and ≥0.75, excellent (32). Given the small periocular measurements, we set the minimum error threshold for the mean absolute difference (MAD) and technical error of measurement (TEM) to 1 unit (millimeter or degree). Relative error of measurement (REM) and relative TEM (rTEM) values can be classified into five categories (excellent, <1%; very good, 1–3.9%; good, 4–6.9%; moderate, 7–9.9%; and poor, >10%) based on the scale proposed by Camison et al. and Andrade et al. (5, 33).

## Results

The measurement results (means and standard error, SD) of the M3 and H2 system are shown in Table 3. Repeatability parameters (ICC, MAD, TEM, REM, and rTEM) within and between the VECTRA M3 and H2 devices are presented in Tables 4, 5.

**Table 3:** Means and standard deviations (SDs) of all measurements (mm or degrees).

**Parameters**	**M3**				**H2**			
	**Capture 1**		**Capture 2**		**Capture 1**		**Capture 2**	
	**Mean**	**SD**	**Mean**	**SD**	**Mean**	**SD**	**Mean**	**SD**
**Liner distances (mm)**								
PFW	29.38	1.76	29.37	1.86	30.05	1.69	30.00	1.68
PFH	12.01	1.28	12.19	1.26	12.28	1.46	12.15	1.49
EEnD_I	16.78	1.95	16.84	2.07	16.46	1.87	16.39	1.72
EEnD_M_	23.81	2.11	23.84	2.11	23.63	2.26	23.58	2.25
EEnD_S_	28.45	2.81	28.55	2.71	28.38	2.91	28.38	2.93
FPDm	4.37	1.16	4.47	1.17	4.83	1.12	4.91	1.13
EPDm_I	13.93	1.95	13.96	2.11	13.94	1.77	13.98	1.71
EPDm_M	21.05	1.97	21.14	2.04	21.32	2.11	21.33	2.09
EPDm_S	25.50	2.49	25.64	2.52	25.83	2.62	25.85	2.59
FLmD	3.96	1.14	3.93	1.16	4.02	0.86	4.04	0.91
ELmD_I	10.88	2.04	10.76	2.17	10.50	1.81	10.60	1.77
ELmD_M	17.75	1.96	17.64	2.15	17.53	2.00	17.65	2.08
ELmD_S	22.23	2.56	22.13	2.69	22.10	2.58	22.21	2.66
FPD	3.39	1.16	3.48	1.18	3.52	0.97	3.71	1.08
EPD_I	10.08	2.01	10.03	1.92	9.43	1.91	9.66	1.83
EPD_M	16.26	1.82	16.28	1.88	15.78	1.98	15.97	2.13
EPD_S	20.83	2.57	20.83	2.70	20.40	2.74	20.59	2.86
FLlD	3.77	1.08	3.80	1.08	3.81	1.02	3.87	0.99
ELlD_I	11.54	2.43	11.50	2.28	10.76	2.23	10.92	2.04
ELlD_M	17.11	1.90	17.06	1.97	16.45	1.80	16.58	1.95
ELlD_S	21.68	2.57	21.63	2.82	21.19	2.71	21.30	2.90
FPDl	4.62	0.94	4.63	0.89	4.89	1.20	4.83	1.04
EPDl_I	13.90	2.78	14.01	2.68	13.48	2.51	13.46	2.38
EPDl_M	19.24	2.14	19.32	2.19	18.80	2.53	18.94	2.03
EPDl_S	23.75	2.78	23.83	2.99	23.57	2.75	23.64	2.94
FExD	6.93	1.05	6.89	1.06	7.42	1.47	7.29	1.47
EExD_I	17.88	3.09	18.01	3.11	17.69	2.91	17.58	2.89
EExD_M	23.07	2.35	23.21	2.44	22.96	2.14	22.93	2.14
EExD_S	27.13	2.92	27.30	2.97	27.09	2.71	27.13	2.79
FExDl	4.77	1.17	4.61	1.18	4.31	1.04	4.32	1.02
EExDl_I	14.77	3.21	14.73	3.21	13.65	3.13	13.49	3.03
EExDl_M	19.44	2.58	19.42	2.57	18.60	2.42	18.50	2.38
EExDl_S	23.38	3.19	23.43	3.13	22.71	3.03	22.63	3.04
ID	11.91	0.49	11.97	0.49	11.90	0.46	11.86	0.44
EnD^*^	32.45	2.60	32.43	2.71	32.40	2.72	32.44	2.75
PD^*^	62.71	3.24	62.72	3.26	62.39	3.14	62.27	3.19
ExD^*^	89.93	4.29	90.04	4.22	90.91	4.28	90.87	4.27
**Curvatures (mm)**								
UPML	38.01	2.75	38.25	2.62	39.03	2.64	38.61	2.55
UPMLm	25.32	2.22	25.46	2.12	26.32	2.18	26.06	1.89
LPML	33.60	2.34	33.75	2.20	34.28	2.41	34.17	2.52
LPMLm	23.20	1.95	23.30	1.71	24.23	1.97	24.19	1.97
EL_I	59.14	5.63	59.22	5.43	59.30	5.64	59.39	5.30
EL_M	70.96	9.28	71.19	9.17	71.37	8.74	71.49	8.55
EL_S	68.91	8.86	69.16	8.84	69.52	8.35	69.59	8.31
**Angles (** **°** **)**								
MCA	41.79	4.45	42.38	4.07	43.04	4.52	42.80	4.78
MCAm	61.65	9.36	62.03	8.71	59.79	9.40	59.07	9.54
LCA	40.93	4.66	41.36	4.63	39.70	4.79	39.55	4.78
LCAm	69.64	10.56	70.65	10.59	62.82	9.44	62.64	9.30
CT	168.33	3.53	168.18	3.44	167.19	3.62	167.13	3.67

* *N = 60; for the rest, N = 120; SD, standard deviations*.

**Table 4:** Intra-device reliability results of VECTRA M3 and H2 for periocular measurements.

**Device comparison**	**M3 vs. M3**						**H2 vs. H2**					
	**ICC (CI 95%)**	**MAD**	**TEM**	**rTEM**	**REM**	***p*-value**	**ICC (CI 95%)**	**MAD**	**TEM**	**rTEM**	**REM**	***p*-value**
**Liner distances (mm)**												
PFW	0.84 (0.77–0.88)	0.02	0.74	2.51	0.06	0.948	0.95 (0.93–0.96)	0.05	0.38	1.27	0.16	0.332
PFH	0.86 (0.79–0.90)	0.19	0.49	4.03	1.56	**<0.001** ^†^	0.87 (0.82–0.91)	0.12	0.54	4.42	1.01	0.078
EEnD_I	0.89 (0.85–0.93)	0.05	0.66	3.91	0.31	0.544	0.92 (0.89–0.94)	0.06	0.51	3.09	0.38	0.349
EEnD_M_	0.90 (0.86–0.93)	0.04	0.68	2.85	0.16	0.665	0.97 (0.96–0.98)	0.05	0.39	1.64	0.22	0.305
EEnD_S_	0.92 (0.89–0.95)	0.10	0.77	2.68	0.34	0.331	0.98 (0.97–0.99)	0.00	0.41	1.45	0.00	0.996
FPDm	0.86 (0.81–0.90)	0.10	0.44	9.88	2.27	0.075	0.89 (0.85–0.92)	0.08	0.37	7.65	1.67	0.062^†^
EPDm_I	0.88 (0.83–0.91)	0.03	0.71	5.11	0.20	0.765	0.91 (0.87–0.94)	0.05	0.52	3.75	0.32	0.508
EPDm_M	0.91 (0.87–0.93)	0.09	0.62	2.94	0.43	0.259	0.96 (0.94–0.97)	0.01	0.43	2.02	0.06	0.848
EPDm_S	0.93 (0.91–0.95)	0.14	0.65	2.53	0.54	0.100	0.98 (0.97–0.98)	0.03	0.40	1.56	0.10	0.622
FLmD	0.85 (0.79–0.89)	0.02	0.45	11.28	0.60	0.681	0.78 (0.70–0.84)	0.01	0.42	10.34	0.27	0.860
ELmD_I	0.89 (0.85–0.92)	0.12	0.70	6.49	1.12	0.183	0.88 (0.83–0.91)	0.10	0.64	6.02	0.92	0.237
ELmD_M	0.89 (0.84–0.92)	0.11	0.70	3.97	0.62	0.228	0.91 (0.87–0.94)	0.11	0.61	3.49	0.64	0.160
ELmD_S	0.92 (0.88–0.94)	0.09	0.77	3.47	0.42	0.540^†^	0.94 (0.92–0.96)	0.11	0.65	2.92	0.47	0.211
FPD	0.86 (0.81–0.90)	0.09	0.44	12.76	2.58	0.091^†^	0.70 (0.59–0.78)	0.19	0.57	15.76	5.16	0.110
EPD_I	0.90 (0.87–0.93)	0.04	0.61	6.07	0.43	0.586	0.79 (0.71–0.85)	0.22	0.86	8.98	2.32	**0.044**
EPD_M	0.88 (0.83–0.92)	0.02	0.64	3.95	0.10	0.854	0.84 (0.77–0.88)	0.19	0.84	5.30	1.19	0.810
EPD_S	0.91 (0.88–0.94)	0.01	0.78	3.75	0.03	0.956	0.92 (0.89–0.95)	0.19	0.78	3.81	0.93	0.057
FLlD	0.83 (0.77–0.88)	0.04	0.45	11.76	0.97	0.242^†^	0.64 (0.53–0.74)	0.07	0.60	15.72	1.68	0.468^†^
ELlD_I	0.92 (0.89–0.94)	0.03	0.67	5.84	0.30	0.695	0.87 (0.81–0.91)	0.16	0.78	7.23	1.51	0.107
ELlD_M	0.87 (0.81–0.90)	0.06	0.71	4.17	0.32	0.550	0.84 (0.78–0.89)	0.13	0.75	4.52	0.77	0.185
ELlD_S	0.91 (0.87–0.94)	0.05	0.82	3.80	0.21	0.676	0.93 (0.90–0.95)	0.11	0.77	3.62	0.52	0.268
FPDl	0.73 (0.63–0.80)	0.01	0.48	10.31	0.29	0.828	0.73 (0.64–0.81)	0.06	0.59	12.04	1.23	0.430
EPDl_I	0.94 (0.91–0.96)	0.10	0.68	4.86	0.74	0.241	0.94 (0.91–0.96)	0.02	0.61	4.56	0.12	0.840
EPDl_M	0.90 (0.86–0.93)	0.08	0.68	3.53	0.40	0.380	0.67 (0.56–0.76)	0.14	1.33	7.03	0.73	0.421
EPDl_S	0.92 (0.89–0.95)	0.08	0.80	3.35	0.32	0.463	0.95 (0.93–0.97)	0.07	0.63	2.68	0.31	0.373
FExD	0.81 (0.73–0.86)	0.05	0.47	6.73	0.69	0.427	0.83 (0.77–0.88)	0.13	0.60	8.17	1.74	0.169^†^
EExD_I	0.96 (0.94–0.97)	0.12	0.65	3.63	0.67	0.152	0.97 (0.96–0.98)	0.11	0.51	2.90	0.61	0.105
EExD_M	0.93 (0.91–0.95)	0.14	0.62	2.70	0.62	0.077	0.95 (0.93–0.96)	0.03	0.49	2.13	0.15	0.591
EExD_S	0.94 (0.91–0.96)	0.16	0.74	2.71	0.60	0.086	0.96 (0.95–0.97)	0.04	0.55	2.01	0.14	0.587
FExDl	0.70 (0.59–0.80)	0.16	0.65	13.87	3.51	0.050	0.79 (0.71–0.85)	0.00	0.48	11.03	0.07	0.960
EExDl_I	0.96 (0.95–0.98)	0.03	0.61	4.15	0.22	0.684	0.96 (0.95–0.98)	0.16	0.59	4.32	1.15	**0.039**
EExDl_M	0.95 (0.93–0.96)	0.02	0.60	3.06	0.10	0.801	0.95 (0.93–0.97)	0.10	0.53	2.86	0.54	0.144
EExDl_S	0.94 (0.92–0.96)	0.05	0.76	3.24	0.22	0.609	0.96 (0.94–0.97)	0.08	0.63	2.76	0.34	0.341
ID	0.83 (0.76–0.88)	0.06	0.21	1.74	0.47	**0.037**	0.90 (0.85–0.93)	0.03	0.15	1.22	0.27	0.085
EnD*	0.99 (0.90–1.00)	0.02	0.15	0.66	0.05	0.457^†^	0.99 (0.99–1.0)	0.04	0.21	0.66	0.12	0.369^†^
PD*	1.00 (1.00–1.00)	0.00	0.12	0.26	0.01	0.921	0.96 (0.94–0.98)	0.12	0.62	1.00	0.19	0.298
ExD*	0.99 (0.98–0.99)	0.11	0.37	0.42	0.13	0.241	0.99 (0.98–0.99)	0.04	0.52	0.57	0.04	0.692
**Curvatures (mm)**												
UPML	0.84 (0.78–0.88)	0.24	0.93	2.85	0.56	0.092	0.83 (0.75–0.88)	0.42	1.09	2.81	1.08	**0.002**
UPMLm	0.82 (0.75–0.87)	0.14	0.59	1.74	0.45	0.238	0.78 (0.70–0.84)	0.26	0.97	3.69	0.98	0.141^†^
LPML	0.93 (0.91–0.95)	0.15	0.67	1.67	0.41	**0.042**	0.90 (0.86–0.93)	0.11	0.78	2.27	0.31	0.286
LPMLm	0.87 (0.81–0.91)	0.10	1.08	1.82	0.13	0.276	0.93 (0.90–0.95)	0.04	0.54	2.24	0.16	0.577
EL_I	0.96 (0.95–0.97)	0.08	1.52	2.15	0.32	0.585	0.94 (0.92–0.96)	0.09	1.32	2.22	0.15	0.617
EL_M	0.97 (0.96–0.98)	0.22	1.32	2.59	0.37	0.354^†^	0.99 (0.98–0.99)	0.12	1.01	1.42	0.17	0.345
EL_S	0.98 (0.97–0.99)	0.25	1.75	4.15	0.37	0.138	0.99 (0.98–0.99)	0.08	0.92	1.33	0.11	0.513
**Angles (** **°** **)**												
MCA	0.84 (0.77–0.88)	0.58	1.90	4.61	1.05	**0.009**	0.86 (0.81–0.90)	0.23	1.74	4.05	0.55	0.298
MCAm	0.86 (0.80–0.90)	0.38	6.05	8.63	1.43	0.384	0.90 (0.85–0.93)	0.72	3.09	5.20	1.21	0.071
LCA	0.84 (0.77–0.88)	0.43	1.19	6.39	0.09	0.077	0.87 (0.82–0.91)	0.15	1.74	4.39	0.38	0.506
LCAm	0.68 (0.57–0.76)	1.00	0.74	13.16	0.06	0.201	0.82 (0.75–0.87)	0.18	4.03	6.42	0.29	0.730
CT	0.88 (0.84–0.92)	0.15	0.49	1.02	1.56	0.335	0.95 (0.92–0.96)	0.06	0.85	0.51	0.03	0.606
Mean	0.89	0.13	1.02	5.51	0.61		0.89	0.12	0.80	4.43	0.68	

*CI, confidence interval*.

† *Represents p-values calculated from Wilcoxon's signed-rank test and the rest derived from paired-samples t-test*.

*Results with P <0.05 are marked in bold*.

**Table 5:** Inter-device reliability results of VECTRA M3 and H2 for periocular measurements.

**Device comparison**	**M3 vs. H2**						**M2 vs. H2 (Mean)**					
	**ICC (CI 95%)**	**MAD**	**TEM**	**rTEM**	**REM**	***p*-value**	**ICC (CI 95%)**	**MAD**	**TEM**	**rTEM**	**REM**	***p*-value**
**Liner distances (mm)**												
PFW	0.83 (0.46–0.93)	0.67	0.73	2.46	2.25	**<0.001**	0.82 (0.51–0.91)	0.65	0.76	2.55	2.20	**<0.001**
PFH	0.80 (0.70–0.86)	0.27	0.63	5.18	2.23	**0.001**	0.86 (0.81–0.90)	0.12	0.50	4.07	0.94	0.073
EEnD_I	0.79 (0.71–0.85)	0.33	0.88	5.31	1.98	**0.004**	0.85 (0.76–0.90)	0.39	0.73	4.39	2.32	**<0.001**
EEnD_M	0.88 (0.83–0.91)	0.18	0.78	3.27	0.74	0.080	0.92 (0.88–0.95)	0.22	0.61	2.58	0.93	**0.005**
EEnD_S	0.90 (0.87–0.93)	0.07	0.89	3.13	0.24	0.549	0.95 (0.93–0.96)	0.12	0.64	2.27	0.41	0.160
FPDm	0.66 (0.45–0.78)	0.45	0.69	15.10	9.86	**<0.001**	0.72 (0.48–0.84)	0.44	0.61	13.17	9.56	**<0.001**
EPDm_I	0.81 (0.73–0.86)	0.01	0.82	5.91	0.05	0.951	0.85 (0.79–0.90)	0.02	0.71	5.09	0.11	0.869
EPDm_M	0.86 (0.80–0.90)	0.27	0.76	3.60	1.26	**0.006**	0.90 (0.85–0.98)	0.23	0.66	3.11	1.08	**0.007**
EPDm_S	0.89 (0.84–0.92)	0.33	0.85	3.33	1.27	**0.003**	0.92 (0.88–0.95)	0.27	0.73	2.83	1.05	**0.004**
FLmD	0.59 (0.46–0.70)	0.07	0.45	11.15	1.65	0.432	0.68 (0.57–0.77)	0.08	0.56	13.97	2.09	0.249
ELmD_I	0.77 (0.67–0.84)	0.38	0.94	8.77	3.53	^†^	0.80 (0.72–0.86)	0.27	0.86	8.05	2.51	**0.005** ^^†^^
ELmD_M	0.80 (0.73–0.86)	0.22	0.89	5.02	1.24	0.056	0.84 (0.78–0.88)	0.11	0.81	4.58	0.61	0.304
ELmD_S	0.85 (0.79–0.89)	0.12	0.99	4.48	0.55	0.346	0.88 (0.83–0.92)	0.02	0.90	4.04	0.10	0.848
FPD	0.69 (0.59–0.78)	0.13	0.60	17.29	3.86	0.108^†^	0.73 (0.63–0.80)	0.18	0.55	15.61	5.18	**0.011** ^^†^^
EPD_I	0.71 (0.54–0.81)	0.64	1.09	11.15	6.60	**<0.001** ^^†^^	0.76 (0.63–0.84)	0.51	0.92	9.39	5.22	**<0.001** ^^†^^
EPD_M	0.71 (0.58–0.79)	0.48	1.05	6.56	2.99	**<0.001**	0.77 (0.67–0.84)	0.39	0.91	5.68	2.44	**0.001**
EPD_S	0.81 (0.73–0.87)	0.43	1.16	5.65	2.08	**0.004**	0.85 (0.79–0.89)	0.34	1.04	5.04	1.62	**0.012**
FLlD	0.63 (0.51–0.73)	0.04	0.64	16.85	1.00	0.962^†^	0.73 (0.63–0.80)	0.05	0.51	13.42	1.36	0.552^†^
ELlD_I	0.83 (0.60–0.91)	0.78	1.00	8.98	6.97	**<0.001**	0.84 (0.64–0.92)	0.68	0.89	7.99	6.07	**<0.001**
ELlD_M	0.74 (0.54–0.84)	0.66	0.98	5.86	3.93	**<0.001**	0.78 (0.61–0.87)	0.57	0.88	5.25	3.38	**<0.001**
ELlD_S	0.84 (0.76–0.89)	0.48	1.06	4.94	2.26	**<0.001**	0.86 (0.80–0.90)	0.41	1.02	4.75	1.89	**0.002**
FPDl	0.45 (0.29–0.58)	0.27	0.82	17.27	5.68	0.074^†^	0.56 (0.42–0.68)	0.23	0.64	13.52	4.92	**0.004**
EPDl_I	0.88 (0.82–0.92)	0.43	0.93	6.76	3.10	**<0.001**	0.88 (0.80–0.93)	0.49	0.89	6.51	3.53	**<0.001** ^^†^^
EPDl_M	0.60 (0.46–0.70)	0.44	1.51	7.94	2.32	**0.001** ^^†^^	0.74 (0.64–0.81)	0.41	1.09	5.71	2.15	**0.003**
EPDl_S	0.88 (0.83–0.91)	0.18	0.97	4.09	0.78	0.143	0.89 (0.85–0.92)	0.19	0.93	3.92	0.78	0.124
FExD	0.45 (0.28–0.59)	0.49	0.98	13.63	6.80	**0.004**	0.53 (0.36–0.66)	0.45	0.87	12.15	6.28	**<0.001**
EExD_I	0.92 (0.89–0.94)	0.20	0.86	4.85	1.11	0.077	0.94 (0.90–0.96)	0.31	0.76	4.29	1.75	**0.001**
EExD_M	0.90 (0.86–0.93)	0.11	0.72	3.11	0.46	0.255	0.91 (0.87–0.94)	0.19	0.68	2.93	0.84	**0.025**
EExD_S	0.90 (0.86–0.93)	0.05	0.88	3.26	0.17	0.686	0.91 (0.88–0.94)	0.11	0.84	3.08	0.40	0.315
FExDl	0.64 (0.42–0.77)	0.46	0.69	15.15	10.03	**<0.001** ^†^	0.70 (0.51–0.81)	0.37	0.59	13.04	8.26	**<0.001**
EExDl_	0.89 (0.43–0.96)	1.12	1.11	7.80	7.85	**<0.001**	0.89 (0.29–0.96)	1.18	1.09	7.71	8.31	**<0.001**
EExDl_M	0.87 (0.55–0.95)	0.84	0.61	3.21	4.41	**<0.001**	0.88 (0.43–0.96)	0.88	0.88	4.64	4.63	**<0.001**
EExDl_S	0.89 (0.79–0.94)	0.67	1.05	4.58	2.93	**<0.001**	0.90 (0.76–0.95)	0.74	0.97	4.23	3.20	**<0.001**
ID	0.85 (0.79–0.89)	0.02	0.19	1.57	0.15	0.454	0.87 (0.81–0.91)	0.06	0.17	1.39	0.52	**0.003**
EnD*	0.90 (0.98–0.99)	0.05	0.29	0.88	0.14	0.763^†^	0.99 (0.99–1.0)	0.02	0.22	0.66	0.06	0.713^†^
PD*	0.96 (0.93–0.98)	0.33	0.65	1.04	0.52	**0.005**	0.98 (0.92–0.99)	0.39	0.44	0.71	0.62	**<0.001**
ExD*	0.95 (0.65–0.98)	0.98	0.96	1.06	1.08	**<0.001**	0.96 (0.91–0.99)	0.90	0.90	1.00	1.00	**<0.001**
**Curvatures (mm)**												
UPML	0.76 (0.52–0.82)	1.02	1.37	3.56	2.66	**<0.001**	0.82 (0.68–0.89)	0.70	1.09	2.84	1.81	**<0.001**
UPMLm	0.65 (0.39–0.79)	1.00	1.38	5.34	3.87	**<0.001**	0.73 (0.48–0.85)	0.80	1.09	4.21	3.10	**<0.001**
LPML	0.87 (0.69–0.93)	0.68	1.11	3.28	1.99	**<0.001**	0.87 (0.75–0.92)	0.55	0.85	2.51	1.61	**<0.001**
LPMLm	0.72 (0.27–0.87)	1.03	1.12	4.71	4.34	**<0.001**	0.75 (0.24–0.89)	0.96	1.00	4.21	4.05	**<0.001**
EL_I	0.89 (0.84–0.92)	0.16	1.89	3.18	0.27	0.519	0.92 (0.89–0.95)	0.16	1.51	2.54	0.27	0.406
EL_M	0.96 (0.94–0.97)	0.41	1.80	2.52	0.57	**0.048** ^^†^^	0.97 (0.95–0.98)	0.36	1.65	2.32	0.50	0.096
EL_S	0.95 (0.92–0.96)	0.61	2.02	2.93	0.88	**0.020**	0.96 (0.94–0.97)	0.52	1.78	2.57	0.75	**0.023**
**Angles (** **°** **)**												
MCA	0.77 (0.64–0.85)	1.24	2.18	5.14	2.93	**<0.001**	0.84 (0.75–0.89)	0.84	1.75	4.12	1.96	**<0.001**
MCAm	0.79 (0.70–0.86)	1.86	4.32	7.11	3.06	**0.001**	0.83 (0.69–0.90)	2.41	3.76	6.20	3.98	**<0.001**
LCA	0.71 (0.58–0.80)	1.22	2.61	6.49	3.04	**<0.001**	0.77 (0.58–0.86)	1.52	2.26	5.59	3.75	**<0.001**
LCAm	0.39 (0.11–0.58)	6.83	8.73	13.18	10.31	**<0.001**	0.47 (0.04–0.70)	7.42	7.81	11.76	11.17	**<0.001**
CT	0.79 (0.66–0.88)	1.14	1.68	1.00	0.68	**<0.001**	0.84 (0.63–0.91)	1.09	1.46	0.87	0.65	**<0.001**
Mean	0.79	0.63	1.31	7.62	2.83		0.83	0.62	1.10	5.57	2.69	

*CI, confidence interval*.

† *Represents p-values calculated from Wilcoxon's signed-rank test and the rest derived from paired-samples t-test. Results with P < 0.05 are marked in bold*.

### Intra-Device Reliability With VECTRA M3

**Figure 3:**
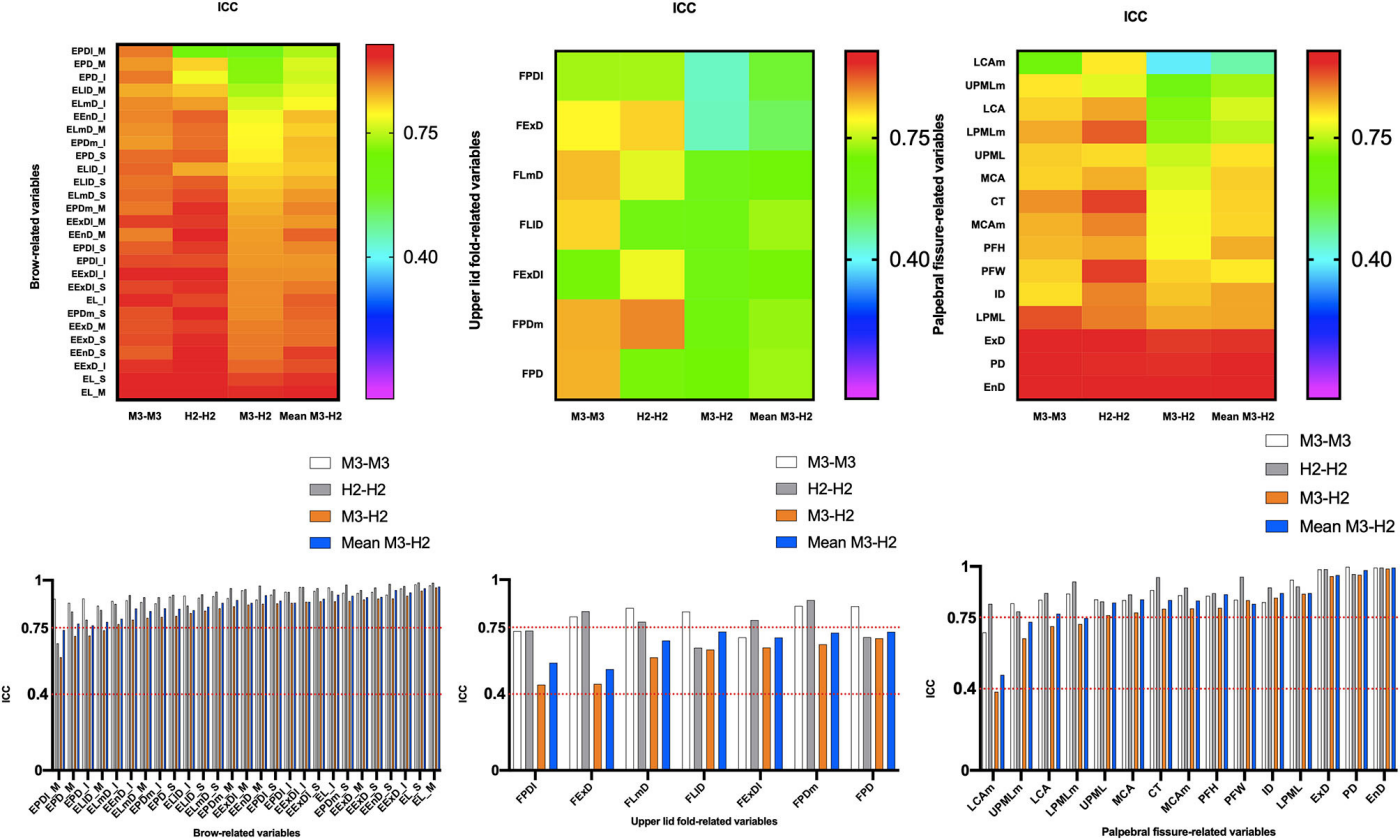
Intra- and inter-device of intraclass correlation coefficient (ICC) for all periocular region measurements of 3D images. ICC values allowed the classification of the agreement into three classes: <0.4, poor agreement; 0.4–0.75, satisfactory; and ≥0.75, excellent.

**Figure 4:**
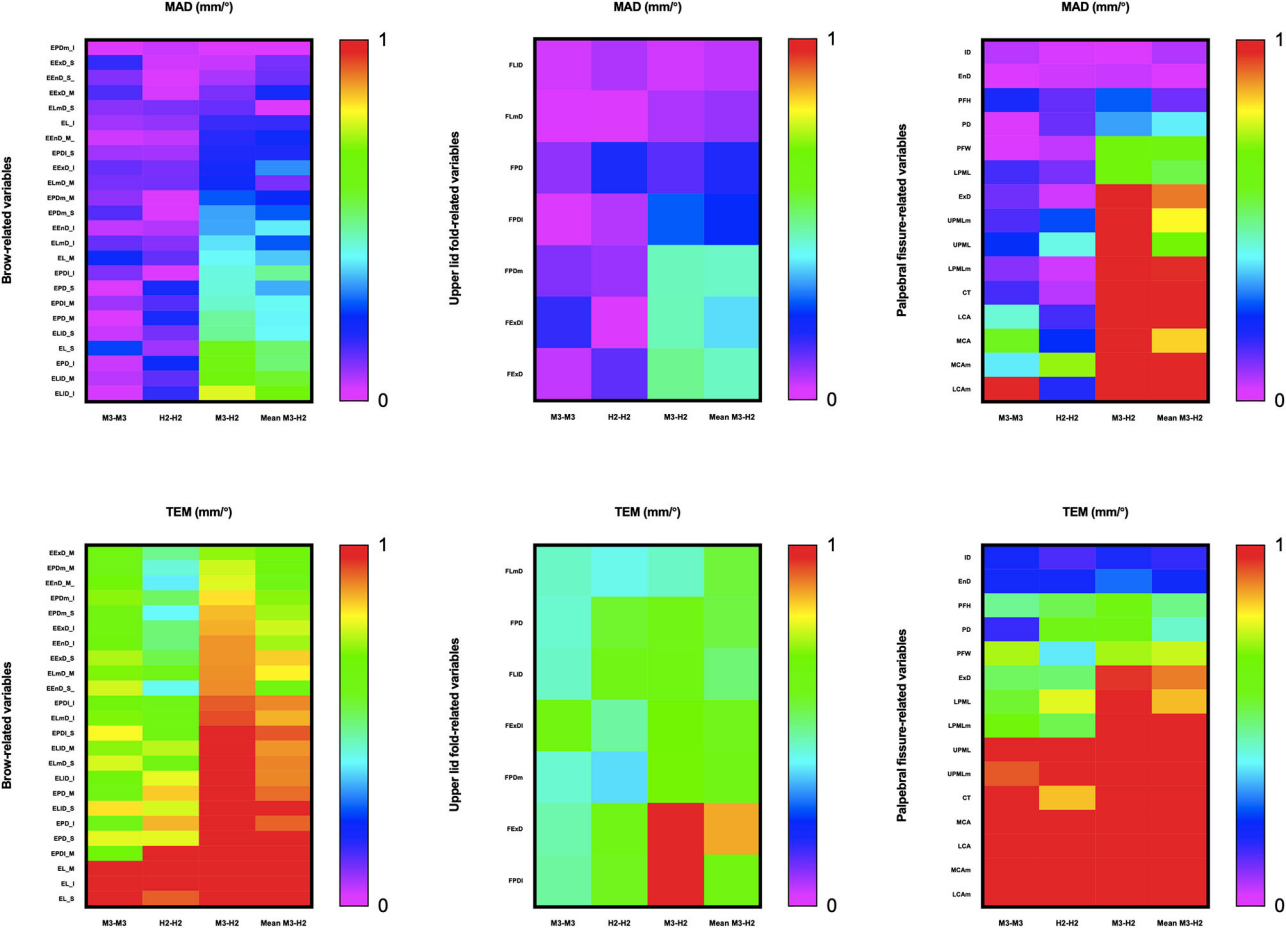
Intra- and inter-device mean absolute difference (MAD) and technical error of measurement (TEM) for periocular measurements on all 3D images. The acceptable error threshold is set to 1 unit.

**Figure 5:**
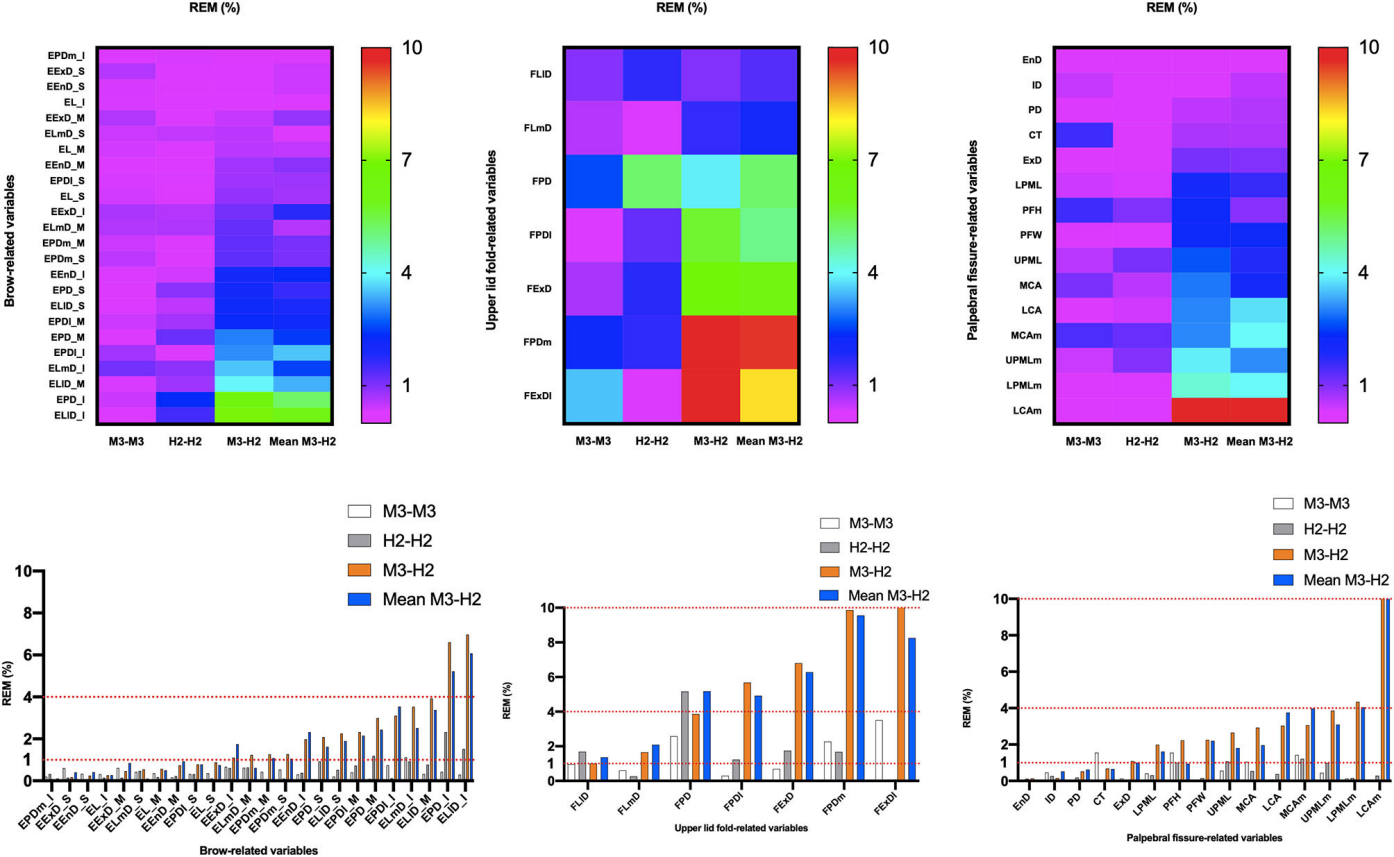
Intra- and inter-device relative error of measurement (REM) for all periocular region measurements of 3D images. Reliability category criteria were as follows: excellent, <1%; very good, 1–3.9%; good, 4–6.9%; moderate, 7–9.9%; and poor, >10%.

**Figure 6:**
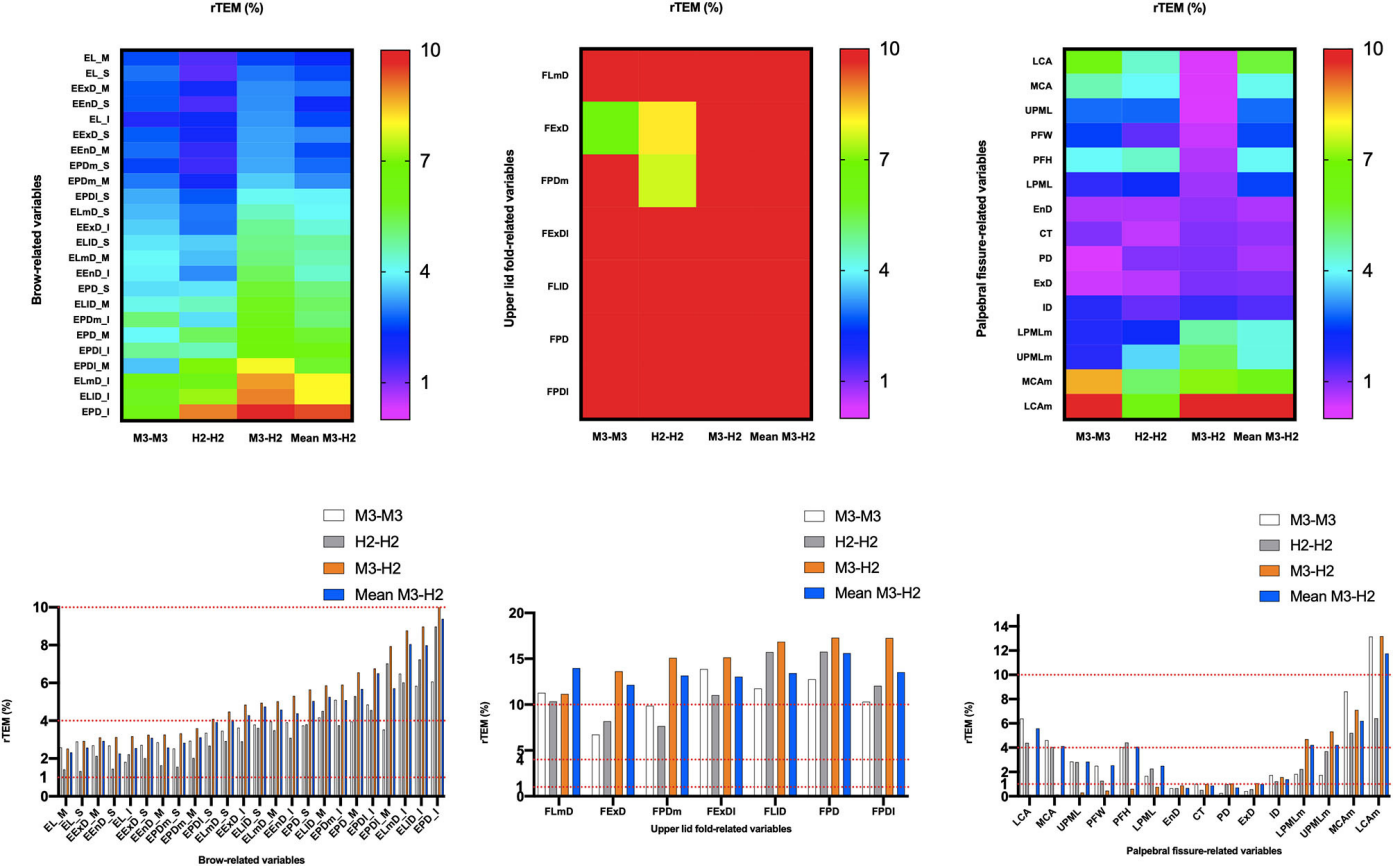
Intra- and inter-device of relative technical error of measurement (rTEM) for all periocular region measurements of 3D images. Reliability category criteria were as follows: excellent, <1%; very good, 1–3.9%; good, 4–6.9%; moderate, 7–9.9%; and poor, >10%.

ICC (Table 4, Figure 3) estimates for most M3 intra-device comparisons were excellent (0.81–1.00), except for two upper lid fold-related variables (FPDI: 0.73, FExDI: 0.70) and one eyelid fissure-related variable (LCAm: 0.68). As shown in Table 6 and Figure 4, MAD was <1 unit for 48 of the 49 parameters, and LCAm was between 1 and 2 units. TEM was <1 unit for 42 parameters (87.5% eyebrow-related variables, 100% upper lid fold-related variables, and 60% palpebral fissure-related variables); the largest measurement error was for the palpebral fissure-related variable MCAm (6.05°). The REM and rTEM results for each comparison are shown in Figure 5 and Figure 6. Of seven upper lid fold-related variables, 57.1% of REM values were <1 and 42.9% of values were between 1 and 3.9%. As for rTEM, 71.4% of variables were >10%, except for FExD, which was <7%, and FDPm, which was <10%. FExDI (rTEM = 13.87%) had the largest value. REM was <1% for 73.3% of palpebral fissure-related variables, and 26.7% were in the 1–3.9% range. Except for MCAm (rTEM = 8.63%) and LCAm (rTEM = 13.61%), 86.7% of palpebral fissure-related values showed an rTEM <7% (20% of variables were <1%, 46.7% were between 1 and 3.9%, and 20% were between 4 and 6.9%). Moreover, the rTEM and REM of all brow-related variables were <7%. Additionally, 95.8% of REM values were <1%, 75% of rTEM and 4.2% of REM values were between 1 and 3.9%, and 25% of rTEM values were between 4 and 6.9%.

**Table 6:** Percentage of different periocular measurement variables in each reliability rating classification for VECTRA M3 and H2.

**Variables**	**Upper lid fold-related variables (7/7)**				**Palpebral fissure-related variables (15/15)**				**Eyebrow-related variables (24/24)**			
	**M3-M3**	**H2-H2**	**M3-H2**	**M3-H2 (Mean)**	**M3-M3**	**H2-H2**	**M3-H2**	**M3-H2 (Mean)**	**M3-M3**	**H2-H2**	**M3-H2**	**M3-H2 (Mean)**
**ICC**												
Excellent (≥0.75)	71.4% (5/7)	57.1% (4/7)	0% (0/7)	0% (0/7)	93.3% (14/15)	100% (15/15)	73.3% (11/15)	86.7% (13/15)	100% (24/24)	95.8% (23/24)	83.3% (20/24)	95.8% (23/24)
Satisfactory (0.4–0.75)	28.6% (2/7)	42.9% (3/7)	100% (7/7)	100% (7/7)	6.7% (1/15)	0% (0/15)	20% (3/15)	13.3% (2/15)	0% (0/24)	4.2% (1/24)	16.7% (4/24)	4.2% (1/24)
Poor (<0.4)	0% (0/7)	0% (0/7)	0% (0/7)	0% (0/7)	0% (0/15)	0% (0/15)	6.7% (1/15)	0% (0/15)	0% (0/24)	0% (0/24)	0% (0/24)	0% (0/24)
**MAD**												
<1 unit	100% (7/7)	100% (7/7)	100% (7/7)	100% (7/7)	93.3% (14/15)	100% (15/15)	53.3% (8/15)	73.3% (11/15)	100% (24/24)	100% (24/24)	100% (24/24)	100% (24/24)
>1 unit	–	–	–	–	6.7% (1/15)	–	46.7% (7/15)	26.7 (4/15)	–	–	–	–
**TEM**												
<1 unit	100% (7/7)	100% (7/7)	85.7% (6/7)	100% (7/7)	60% (9/15)	60% (9/15)	40% (6/15)	46.7% (7/15)	87.5% (21/24)	87.5% (21/24)	62.5% (15/24)	75% (18/24)
>1 unit	–	–	14.3% (1/7)	–	40% (6/15)	40% (6/15)	60% (9/15)	53.3% (8/15)	12.5% (3/24)	12.5% (3/24)	37.5% (9/24)	25% (6/24)
**REM**												
Excellent (<1%)	57.1% (4/7)	28.6% (2/7)	–	–	73.3% (11/15)	80% (12/15)	26.7% (4/15)	33.3% (5/15)	95.8% (23/24)	87.5% (21/24)	41.7% (10/24)	45.8% (11/24)
Very good (1–3.9%)	42.9% (3/7)	57.1% (4/7)	42.9% (3/7)	28.6% (2/7)	26.7% (4/15)	20% (3/15)	60% (9/15)	46.7% (7/15)	4.2% (1/24)	12.5% (3/24)	50% (12/24)	45.8% (11/24)
Good (4–6.9%)	0% (0/7)	14.3% (1/7)	28.6% (2/7)	42.9% (3/7)	–	–	6.7% (1/15)	6.7% (1/15)	–	–	4.2% (1/24)	8.3% (2/24)
Moderate (7–9.9%)	–	–	14.3% (1/7)	28.6% (2/7)	–	–	–	–	–	–	4.2% (1/24)	–
Poor (>10%)	–	–	14.3% (1/7)	–	–	–	6.7% (1/15)	6.7% (1/15)	–	–	–	–
**rTEM**												
Excellent (<1%)	–	–	–	–	20% (3/15)	26.7% (4/15)	46.7% (7/15)	26.7% (4/15)	–	–	–	
Very good (1–3.9%)	–	–	–	–	46.7% (7/15)	40% (6/15)	26.7% (4/15)	26.7% (4/15)	75% (18/24)	70.8% (17/24)	37.5% (9/24)	41.7% (10/24)
Good (4–6.9%)	14.3% (1/7)	–	–	–	20% (3/15)	33.3% (5/15)	–	40% (6/15)	25% (6/24)	16.7% (4/24)	45.8% (11/24)	45.8% (11/24)
Moderate (7–9.9%)	14.3% (1/7)	28.6% (2/7)	–	–	6.7% (1/15)	–	6.7% (1/15)	–	–	12.5% (3/24)	12.5% (3/24)	12.5% (3/24)
Poor (>10%)	71.4% (5/7)	71.4% (5/7)	100% (7/7)	100% (7/7)	6.7% (1/15)	–	6.7% (1/15)	6.7% (1/15)	–	–	4.2% (1/24)	–

### Intra-Device Reliability With VECTRA H2

Intra-device ICC (Table 4, Figure 3) was above 0.75 for most variables measured by device H2, except for one eyebrow-related parameter (EPDl_M) and three upper lid fold-related variables (FPD, FLl, and FPDl), with an ICC between 0.4 and 0.75. Figure 4 and Table 6 show that all 49 parameters with a MAD and 41 parameters with TEM had a value of <1 unit (87.5% of eyebrow-related variables, 100% of upper lid fold-related variables, and 60% of palpebral fissure-related variables). The largest measurement error was the palpebral fissure-related variable LCAm (4.03°); 28.6% of upper lid fold-related variables showed an REM <1%, 57.1% had variables between 1 and 3.9%, and the maximum value of FPD was 5.163%. All these variables had an rTEM >7%, 28.6% of these variables had an rTEM between 7 and 10%, and 71.4% had an rTEM >10%; the maximum value was FPD (rTEM = 15.76%). All palpebral fissure-related variables showed an REM and rTEM <7% (80% of REM and 26.7% of rTEM values were <1%, 20% of REM and 40% of rTEM values were between 1 and 3.9%, and 33.3% of rTEM values were between 4 and 6.9%). Except for EPDl_M, ELlD_I, and EPD_I with an rTEM >7%, REM and rTEM were <7% across all brow-related variables (87.5% of variables had an REM <1%, 70.8% of rTEM and 12.5% of REM variables were between 1 and 3.9%, and 16.7% of rTEM values were between 4 and 6.9%).

### Inter-Device Reliability Between VECTRA H2 and VECTRA M3

When the first captures of both devices (M3 and H2) were used to compare inter-device reliability; 33 variables showed an ICC (Table 5) >0.75 (83.3% of brow-related variables and 73.3% of palpebral fissure-related variables), 15 variables had ICC values between 0.4 and 0.75 (16.7% of brow-related variables, 100% of upper eyelid fold-related variables, and 20% of palpebral fissure-related variables). The smallest ICC value was for a palpebral fissure-related variable, LCAm (ICC = 0.39). Forty-one variables had an MAD <1 unit (100% of eyebrow-related variables, 100% of upper eyelid fold-related variables, and 53.3% of palpebral fissure-related variables), and the highest MAD value was for LCAm (6.825°). Twenty-eight measurements had a TEM <1 unit (62.5% of brow-related variables, 85.7% of upper eyelid fold-related variables, and 40% of palpebral fissure-related variables), and LCAm (8.731°) had the highest TEM value. Only the rTEM of EnD and 14 variables (including EnD) of REM were <1% (41.7% of eyebrow-related variables and 26.7% of palpebral fissure-related variables; Figure 5). Thirteen variables of rTEM (37.5% of eyebrow-related variables and 26.7% of palpebral fissure-related variables) and 24 variables of REM (50% of brow-related variables, 42.9% of upper eyelid fold-related variables, and 60% of palpebral fissure-related variables) were between 1 and 3.9%, respectively; 13 rTEM (45.8% of brow-related variables and 13.3% of palpebral fissure-related variables) and five REM (37.5% of brow-related variables, 28.6% of upper eyelid fold-related variables, and 6.7% of palpebral fissure-related variables) variables were between 4 and 6.9%; four rTEM (12.5% of eyebrow-related variables and 28.6% of upper eyelid fold-related variables) and one REM (28.6% of upper eyelid fold-related variables) variables were between 7 and 10%. Nine rTEM (4.2% of brow-related variables, 100% of upper eyelid fold-related variables, and 6.7% [1/15] of palpebral fissure-related variables) and two REM values (28.6% [1/7] of upper eyelid fold-related variables and 6.7% of palpebral fissure-related variables) were >10%. The largest rTEM value was FPD (17.29%), while the largest REM value was LCAm (10.31%).

When the mean of two scans for each device was used for comparison, 39 variables had an ICC >0.75, and the remaining 10 variables were between 0.4 and 0.75. Forty-four measurements had a MAD of <1 unit, and 35 measurements had a TEM of <1 unit. Four rTEM and 16 REM values were <1%, 14 rTEM and 21 REM values were between 1 and 3.9%, and 17 rTEM and 6 REM values were between 4 and 6.9%. Three rTEM and two REM values were between 7 and 10%, and eight rTEM and one REM values were >10%. Overall, applying the average of the two captures mildly improved inter-device reliability compared to using only the first capture.

## Discussion

We validated the reliability of portable devices in periocular applications for the first time using a periocular marker developed by Guo et al. (21). The mean results for the intra-device reliability metrics of MAD (0.13 and 0.12 units), REM (0.61 and 0.68%), TEM (1.02 and 0.80 units), rTEM (5.51 and 4.43%), and ICC (0.89 and 0.89) for devices M3 and H2 were highly comparable. For inter-device comparisons, the mean MAD, REM, TEM, rTEM, and ICC were 0.63 units, 2.83%, 1.31 units, 7.62%, and 0.79 units, respectively (0.62 units, 2.69%, 1.10 units, 5.57%, and 0.83 units if the mean values of H2 and M3 were used). Inter-device reliability decreased compared to intra-device reliability and all reliability metrics improved when quoting average values, indicating that we can reduce inter-device variation by using the average of the two captured images when the H2 device is used for photography.

Guo et al. first introduced 52 new periocular landmarks and validated the high reliability of the static VECTRA M3 stereophotogrammetric system for periocular anthropometry (21). The imaging system and landmarks were highly reliable for most measurements. Intra-rater measurements had the highest reliability, followed by inter-rater and intra-device measurements. The results of the M3 intra-device reliability analysis included MAD (0.98 units), REM (4.66%), TEM (0.96 units), rTEM (4.64%), and ICC (0.96). Our results were generally consistent with the aforementioned study, and some indicators were even more reliable.

Several recent studies have validated the reliability of portable stereophotogrammetric devices for facial imaging (5, 31). Camison et al. (5) verified that the portable VECTRA H1 and static 3dMD devices were highly comparable in facial imaging: 136 linear distances had an inter-device mean rTEM value of 1.13% (range, 0.44–2.48%). Fifty-five of these distances (40.4%) were in the “excellent” category (<1%), while the remaining 81 distances (59.6%) were in the “very good” range (<3.9%) (TEM, 0.84 mm). Gibelli et al. (31) and Kim et al. (34) compared the portable VECTRA H1 device with the static VECTRA M3 device in terms of the linear, angular, surface area, and volume measurement for reliability. The results, except for the lip and periocular regions, showed high repeatability for most linear, angular, and surface area measurements in M3 vs. M3, H1 vs. H1, and M3 vs. H1 comparisons (range, 82.2–98.7%; TEM, range, 0.3–2.0 mm, 0.4–1.8 degrees; rTEM, range, 0.2–3.1%). rTEM was primarily classified to provide excellent intra-device and good inter-device comparisons. Notably, they validated the results mainly for the non-periocular regions of the face, thus assessing significant differences in the linear distance and angular type of validation. The current results are generally less reliable than previous studies, possibly due to the eye movements reported in the previous literature (35, 36). Furthermore, the measurements in the periocular region are all small, and previous studies have reported that reliability decreases as measurements decrease (37, 38).

Specifically, the highest reliability was found for most palpebral fissure-related variables in various comparisons, with rTEM primarily categorized as excellent, very good, or good within devices (M3 vs. M3: 0.26–6.39% and H2 vs. H2: 0.51–6.42%). Simultaneously, M3 and H2 comparisons were also excellent, very good, or good (0.02–5.34% and 0.66–6.20%) (first assessment and mean). The next most reliable assessment was for eyebrow-related variables, and within-device rTEM was mainly classified as very good or good (M3 vs. M3: 1.82–6.49% and H2 vs. H2: 1.33–6.02%), while M3 and H2 comparisons were also good (2.52–6.76% and 2.27–6.51%, respectively) (first evaluation and mean). The worst reliability was for the upper eyelid fold-related variables. Within-device rTEM was mainly classified as moderate or poor (M3 vs. M3: 9.88–13.87% and H2 vs. H2: 7.65–15.76%), while M3 compared to H1 (first evaluation and mean) had poor rTEM (11.15–17.29% and 12.15–15.61%, respectively).

TEM and rTEM values were generally consistent and reliable in their respective intra-device comparisons when using M3 and H2 scans for periocular data measurements. In contrast, TEM and rTEM values deteriorated in the M3 vs. H2 comparison. This result may be due to the strong effect of involuntary head and eye movements during acquisition using the H2 device as it requires three consecutive images to be acquired, while the static M3 device acquires the same images simultaneously.

One limitation of the current study comes from the volunteers; only cooperative adults could be invited to participate because it is difficult to ensure that head, eye, and eyelid positions do not shift in children and non-cooperative individuals. Additionally, all data were collected at a fixed location, thus not fully reflecting the portability of the H2 device. Furthermore, the current study involved linear distance, curve, and angle measurements of the periocular region, without measuring its area and volume. Therefore, this study focused on comparing the differences in periocular measurements between healthy Caucasian adults on the two devices and did not include age, race, and patients in the study. Further studies should evaluate the device's reliability in different age groups, different ethnicities, bedside or other indoor settings for patients with limited mobility and the periocular area and volume measurement.

## Conclusions

The intra-device reliability of the two categories of devices in this study was generally consistent, with a slightly poorer inter-device agreement. The palpebral fissure-related variables and eyebrow-related variables had good reliability both within and between devices. This validation study explored the measurement of linear distance, angle, and curve values in the periocular region with the new portable device VECTRA H2, making an essential contribution to validating the VECTRA H2 device in the periocular region. Previous studies used the earlier generation of portable devices, VECTRA H1, and mainly verified the reliability in non-ocular locations of the face. Compared to static devices, portable instruments are relatively inexpensive and location-independent, allowing photography for patients with limited mobility or in remote areas. However, it is disadvantageous in that it has slightly lower reliability than static devices. Therefore, we need to select the most suitable instrument for future clinical applications according to what the actual situation presents.

## Data Availability Statement

The original contributions presented in the study are included in the article/supplementary material, further inquiries can be directed to the corresponding authors.

## Ethics Statement

The studies involving human participants were reviewed and approved by the Ethics Committee of Cologne University. The patients/participants provided their written informed consent to participate in this study. Written informed consent was obtained from the individual(s) for the publication of any potentially identifiable images or data included in this article.

## Author Contributions

WF, AR, and LH: conception, design, and provision of study materials and participants. LH: administrative support. All authors: collection and assembly of data, data analysis and interpretation, and manuscript writing. All authors contributed to the article and approved the submitted version.

## Funding

This study was supported by State Scholarship Fund from the China Scholarship Council (No. 202008080258).

## Conflict of Interest

The authors declare that the research was conducted in the absence of any commercial or financial relationships that could be construed as a potential conflict of interest.

## Publisher's Note

All claims expressed in this article are solely those of the authors and do not necessarily represent those of their affiliated organizations, or those of the publisher, the editors and the reviewers. Any product that may be evaluated in this article, or claim that may be made by its manufacturer, is not guaranteed or endorsed by the publisher.
